# CRL4B^DCAF11^ E3 ligase targets p21 for degradation to control cell cycle progression in human osteosarcoma cells

**DOI:** 10.1038/s41598-017-01344-9

**Published:** 2017-04-26

**Authors:** Zhi Chen, Kun Wang, Canglong Hou, Kaibiao Jiang, Bin Chen, Jianwei Chen, Lifeng Lao, Lie Qian, Guibin Zhong, Zude Liu, Caiguo Zhang, Hongxing Shen

**Affiliations:** 1 Department of Spine Surgery, Renji Hospital, School of Medicine, Shanghai Jiao Tong University, Shanghai, China; 2 Department of Orthopedics, Changhai Hospital, Secondary Military Medical University, Shanghai, China; 3 Department of Dermatology, University of Colorado Anschutz Medical Campus, Aurora, CO USA

## Abstract

Cell cycle progression in mammals is strictly controlled by a number of cyclin-dependent kinases (CDKs) and CDK inhibitors (CKIs), the expression of which is often dysregulated in cancer cells. Our previous work revealed that Cullin 4B (CUL4B), a critical component of the Cullin4B-RING E3 ligase complex (CRL4B), is overexpressed in human osteosarcoma cells through an unknown mechanism. Here, we demonstrated that CUL4B forms an E3 ligase with RBX1 (RING-box 1), DDB1 (DNA damage binding protein 1), and DCAF11 (DDB1 and CUL4 associated factor 11) in human osteosarcoma cells. *In vitro* and *in vivo* ubiquitination analyses indicated that CRL4B^DCAF11^ E3 ligase was able to specifically ubiquitinate a CDK inhibitor—p21^Cip1^ at K16, K154, K161 and K163 but not at K75 and K141. Knocking down any component of the CRL4B^DCAF11^ complex, including CUL4B, DDB1 or DCAF11, using short hairpin RNAs (shRNAs) attenuated the ubiquitination level of p21^Cip1^, inhibited osteosarcoma cell proliferation, led to cell cycle arrest at S phase, and decreased colony formation rate. Taken together, our data suggest that the CRL4B^DCAF11^ complex represents a unique E3 ligase that promotes the ubiquitination of p21^Cip1^ and regulates cell cycle progression in human osteosarcoma cells.

## Introduction

Both prokaryotic and eukaryotic cells are controlled by an ordered series of events known as the cell cycle, which includes the G_0_, G_1_, S, G_2_ and M phases^1, 2^. The cell cycle is strictly controlled by a number of regulatory partner pairs: the cyclins and the cyclin-dependent kinases (CDKs)^3,4.]^ Of these regulatory partners, Cyclin A-CDK2 mainly functions in S phase; Cyclin D-CDK4, Cyclin D-CDK6 and Cyclin E-CDK2 regulate the transition from G_1_ to S phase; and Cyclin B-CDK1 regulates progression from G_2_ to M phase^3, 4^. Cell cycle progression from one phase to the next is controlled by checkpoints, including the G_1_, G_2_/M and metaphase checkpoints^5, 6^. In addition, an effector protein family known as CDK inhibitors (CKIs) also plays important roles in regulating cell cycle progression by suppressing CDK functions^3, 7^. Two families of CKIs, including CDK interacting protein/kinase inhibitory protein (Cip/Kip) and inhibitor of kinase 4/alternative reading frame (INK4a/ARF), are able to disrupt cell cycle progression by affecting different CDKs^8, 9^. For example, members of the Cip/Kip family, including p21, p27 and p57, can suppress CDK2 activity, while members of the INK4a/ARF family, such as INK4A (p16), INK4B (p15), INK4C (p18) and INK4D (p19), are able to inhibit the activities of CDK4 and CDK6^8–10^. Dysregulation of either CDKs or CKIs can disrupt cell cycle progression, thereby resulting in the pathogenesis of a number of diseases, including cancer^10^. Expression of these CDKs and CKIs can be regulated at both the transcriptional and post-transcriptional levels. One example of post-transcriptional regulation is ubiquitination of p21^Cip1^ and p27^Kip^ by different E3 ligases, such as SCF^Skp2^ and CRL4^Cdt2^ ^11–14^.

Eukaryotic organisms contain a family of hydrophobic proteins known as Cullins, which mainly function as scaffolds and which combine with RING proteins and adaptor proteins to form ubiquitin E3 ligase-Cullin-RING ligases (CRLs)^12, 14, 15^. The CRLs recognize different substrates and affect a wide variety of cellular processes, including tumourigenesis^12^. Of particular interest in our studies are the CRL4 E3 ligases, which are formed by Cullin 4 (CUL4), RING-box protein 1 (RBX1), the adaptor protein-damaged DNA binding protein 1 (DDB1), and the DDB1 and CUL4-associated factors (DCAFs)^12, 14, 15^. All of the CRL4s in different organisms share a similar core architecture, in which E3 ligase activity is determined by CUL4-RBX1 and substrate specificity is controlled by DCAFs^12, 14–17^. More than 100 DCAFs have been identified based on characteristic motifs, including WD40 repeats, WDxR motifs, and DDB boxes^18^. The human genome encodes two CUL4 proteins, CUL4A and CUL4B, which share 82% protein sequence identity without showing obvious functional redundancy^17, 18^. *CUL4A* overexpression is widely reported in different cancers, including breast cancer^19^, ovarian cancer^20^, hepatocellular carcinomas^21^, adrenocortical carcinomas^22^, and childhood medulloblastoma^23^, by targeting different substrates such as DDB2, p12, CDT1, STAT1, Chk1 and p21^Cip1^ ^18–23^. In recent years, several studies have determined that *CUL4B* is also overexpressed in some cancer types, such as oesophageal carcinomas and HeLa cells, by targeting H2AK119 and Cyclin E, respectively^24, 25^. Our previous work also identified *CUL4B* overexpression in osteosarcoma cells through an unknown molecular mechanism^26^.

To illuminate the molecular function of CUL4B, especially to determine interacting proteins and to identify the substrates of CRL4B E3 ligase in osteosarcoma cells, we first confirmed interactions between CUL4B and RBX1 or DDB1 *in vivo* and *in vitro*. By examining the protein levels of several DCAFs in osteosarcoma cells, we found that DCAF11 was specifically upregulated, whereas other DCAFs, such as DCAF1, 4, 8, and 15, were not, and we then determined the interactions between DCAF11 and DDB1. By tagging DCAF11 with Flag, followed by immunoprecipitation (IP) and mass spectrometry (MS), we identified proteins that associate with DCAF11. We then identified a specific protein, p21^Cip1^, which interacts with DCAF11. Our *in vivo* and *in vitro* studies support a model in which CRL4B^DCAF11^ E3 targets p21^Cip1^ for ubiquitination to control cell cycle progression in human osteosarcoma cells.

## Results

### CUL4B is upregulated at both the transcriptional and the post-transcriptional levels in human osteosarcoma cells

**Figure 1:**
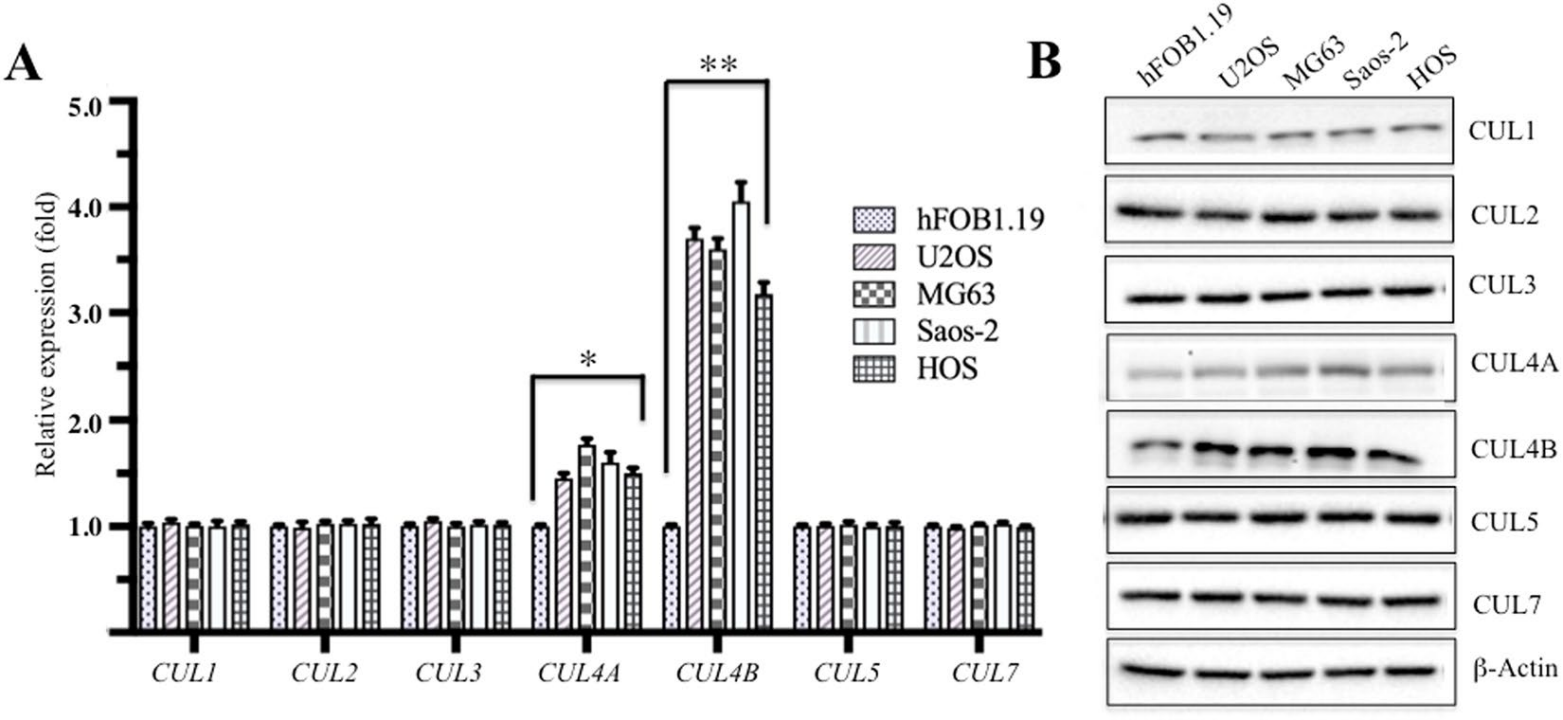
Human osteosarcoma cell lines have elevated *CUL4B* expression. (**A**) *Cullin* mRNA levels, including *CUL1*, *CUL2*, *CUL3*, *CUL4A*, *CUL4B*, *CUL5* and *CUL7*, were determined by qRT-PCR. Expression was normalized to β-Actin in each cell line, and the resulting ratios in hFOB1.19 cells were arbitrarily defined as 1-fold. Representative data from three independent experiments are shown. *P < 0.05 and **P < 0.001. (**B**) WB analyses with specific antibodies against CUL1, CUL2, CUL3, CUL4A, CUL4B, CUL5, CUL7, or β-Actin.

The human genome encodes seven Cullins, CUL1, 2, 3, 4A, 4B, 5, and 7, which function as scaffolds to facilitate the assembly of E3 ligase complexes and transfer ubiquitin from E2 to substrates^12^. Dysregulation of these Cullin members has been broadly reported to contribute to tumourigenesis through diverse mechanisms such as their involvement in DNA damage and repair, cell cycle progression, and the ubiquitination of oncoproteins or tumour suppressors^12^. Our previous work revealed that the *CUL4B* gene was overexpressed in the osteosarcoma cell line Saos-2^26^. However, we did not assess whether only *CUL4B* expression, and not the expression of the other six *Cullin* genes, was upregulated in osteosarcoma cells. To this end, we examined the mRNA and protein levels of these seven *Cullins* in four human osteosarcoma cell lines, U2OS, MG63, Saos-2 and HOS, versus the osteoblast cell line hFOB1.19, a non-cancerous cell line that is commonly used as a control in osteosarcoma studies. Quantitative RT-PCR (qRT-PCR) analysis indicated that *CUL4B* mRNA levels were specifically upregulated (~3–4-fold induction) in these four osteosarcoma cell lines compared with hFOB1.19 cells (Fig. 1A). Among the other six *Cullins*, only *CUL4A* was slightly overexpressed (~1.4–1.7-fold induction) in osteosarcoma cells, but not the other five *Cullins*, including *CUL1*, *CUL2*, *CUL3*, *CUL5* and *CUL7* (Fig. 1A). Consistently, western blot (WB) analysis also indicated that the CUL4B protein was significantly induced (~3–4-fold induction) in all four osteosarcoma cell lines and that CUL4A was slightly overexpressed (~1.3–1.6-fold induction), whereas other Cullin family proteins were not (Fig. 1B, Supplementary Figure 1A). These results suggested that CUL4B is specifically upregulated at both the transcriptional and the post-transcriptional levels in human osteosarcoma cells, implying an important role in the pathogenesis of human osteosarcoma. Given the similar *CUL4B* expression levels in these four osteosarcoma cell lines, we performed the following experiments in only U2OS and/or Saos-2 cells.

### CUL4B interacts with RBX1, DDB1 and DCAF11 to form an E3 complex in human osteosarcoma cells

**Figure 2:**
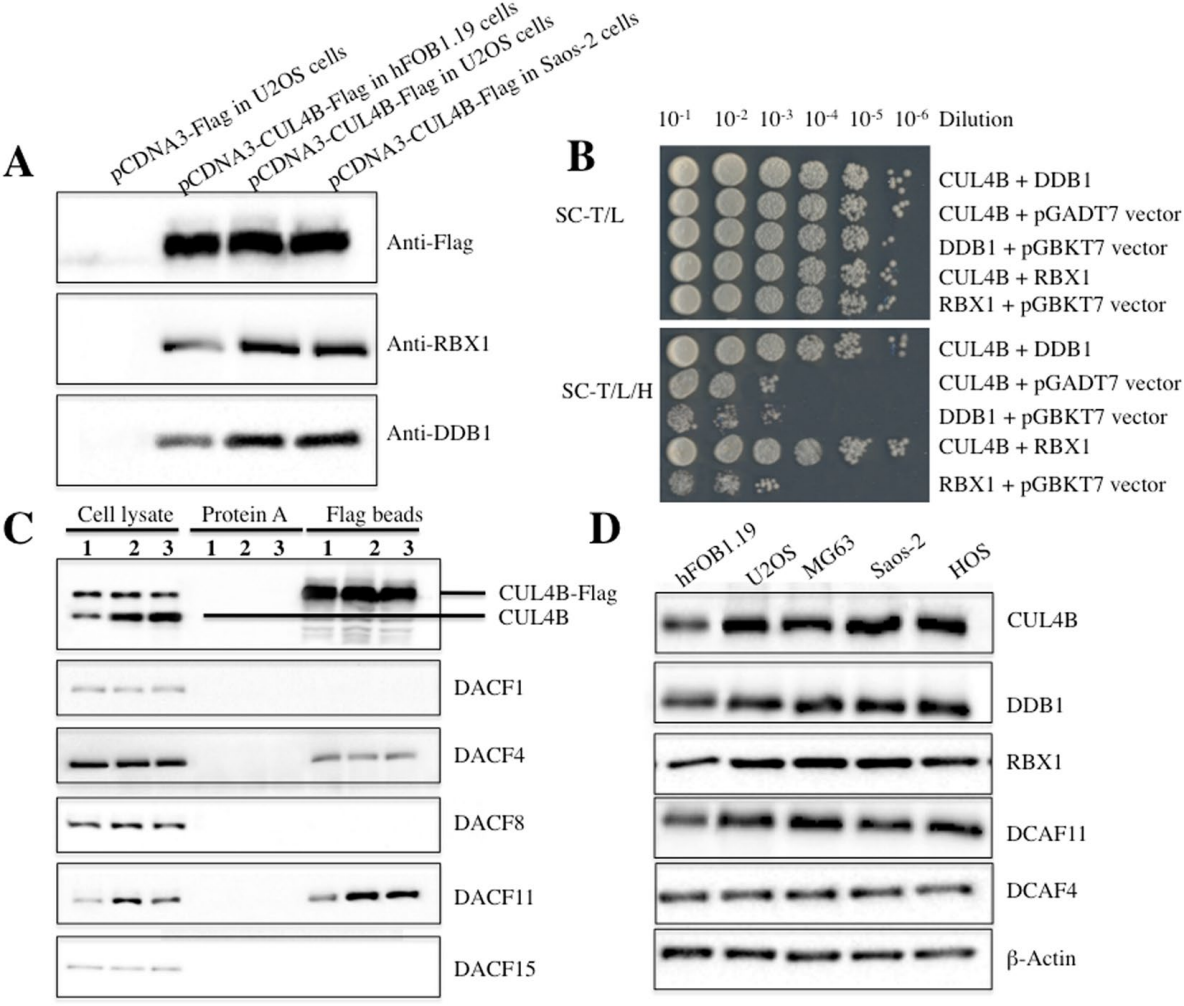
CUL4B interacted with DDB1, RXB1 and DCAF11 to form a complex *in vivo* and *in vitro*. (**A**) Flag-tagged CUL4B interacted with RBX1 and DDB1 *in vivo*. The pCDNA3-CUL4B-Flag or pCDNA3-Flag empty vector was transformed into hFOB1.19, U2OS or Saos-2 cells. After 48 h incubation, Flag-tagged proteins were immunoprecipitated with anti-Flag agarose. The pull-down products were analysed by immunoblotting with anti-Flag, anti-RBX1, or anti-DDB1 antibodies. (**B**) Interactions between CUL4B/DDB1 and CUL4B/RBX1 in yeast. The *pGBKT7-CUL4B* plasmid was co-transformed with *pGADT7-DDB1* or *pGADT7-RBX1* into the yeast strain AH109. Growth of the transformed yeast was assayed on media lacking Trp and Leu (top panel) or lacking Trp, Leu, and His (bottom panel). Columns in each panel represent serial 10-fold dilutions. (**C**) Flag-tagged CUL4B associated with DCAF11 *in vivo*. The pCDNA3-CUL4B-Flag plasmid was transformed into hFOB1.19 (1), U2OS (2) or Saos-2 (3) cells. After 48 h incubation, Flag-tagged proteins were immunoprecipitated with either protein A agarose or anti-Flag-agarose. The cell lysate and pull-down products were analysed by immunoblotting with anti-CUL4B, anti-DCAF1, anti-DCAF4, anti-DCAF8, anti-DCAF11 or anti-DCAF15 antibodies. (**D**) DCAF11 was overexpressed in human osteosarcoma cell lines. Cell lysates were applied to WB analyses with specific antibodies against CUL4B, DDB1, RBX1, DCAF11, DCAF4, or β-Actin.

As previously described^2, 14–17^, CUL4B can associate with RBX1, DDB1 and DCAFs to form complexes, and DCAFs further recognize and ubiquitinate different substrates in a variety of cancer types. To determine whether CUL4B can interact with RBX1 and DDB1 in osteosarcoma cells, we first constructed the *pCDNA3-CUL4B-Flag* vector, which we then transfected into hFOB1.19, U2OS and Saos-2 cells. After IP experiments with anti-Flag-agarose, we detected whether CUL4B pulled down DDB1 and RBX1. As shown in Fig. 2A, the CUL4B-Flag immunocomplex from hFOB1.19, U2OS or Saos-2 cells precipitated not only DDB1, but also RBX1. However, in U2OS cells transfected with the empty vector *pCDNA3-Flag* (negative control), RBX1 and DDB1 failed to immunoprecipitate (Fig. 2A). In addition, the same amount of CUL4B-Flag protein pulled down much more DDB1 and RBX1 protein in U2OS and Saos-2 osteosarcoma cells than in hFOB1.19 cells (Fig. 2A), suggesting that DDB1 and RBX1 are also upregulated in osteosarcoma cells. To further verify the interactions between CUL4B-DDB1 and CUL4B-RBX1, we constructed and transformed the following combinations of plasmids into AH109 yeast cells: *pGBKT7−CUL4B* + *pGADT7−DDB1*, *pGBKT7* + *pGADT7−DDB1*, *pGBKT7−CUL4B* + *pGADT7*, *pGBKT7−CUL4B* + *pGADT7−RBX1*, and *pGBKT7* + *pGADT7−RBX1*. The transformed yeast cells were then plated onto synthetic dropout medium, and positive colonies were selected and assayed to determine these interactions. The yeast two-hybrid (Y2H) results showed that CUL4B also interacts with DDB1 and RBX1 in yeast cells (Fig. 2B). However, under the same growth conditions, CUL4B, DDB1 and RBX1 failed to interact with the corresponding empty vector, including pGADT7 and pGBKT7 (Fig. 2B). Thus, the *in vivo* Co-IP and *in vitro* Y2H studies indicated that CUL4B forms a complex with DDB1 and RBX1.

Thus far, the complex still lacks a DCAF substrate recognition receptor to form a CRL4B E3 ligase. Several DCAF proteins, such as DCAF1, 4, 8, 11 and 15, have been reported to interact with DDB1 in different cancer cells^27–30^. To determine whether any of these proteins could interact with DDB1 in human osteosarcoma cells, the *pCDNA3-CUL4B-Flag* plasmid was transformed into hFOB1.19, U2OS or Saos-2 cells. We then performed IP experiments with anti-Flag-agarose and detected whether CUL4B pulled down DCAF1, 4, 8, 11 and 15. As shown in Fig. 2C, the CUL4B-Flag immunocomplex from hFOB1.19, U2OS or Saos-2 cells pulled down DCAF4 and DCAF11 (Fig. 2C), but not DCAF1, 8 and 15, indicating that these two DCAFs form complexes with CUL4 and DDB1 in osteosarcoma cells. Comparing the DCAF4 and DCAF11 protein levels in cell lysates and in the CUL4B-Flag immunocomplex revealed that DCAF11 was upregulated in the cell lysates of U2OS and Saos-2 compared with hFOB1.19 cells (Fig. 2C), and the same amount of CUL4B-Flag immunocomplex was able to pull down much more DCAF11 protein in osteosarcoma cells than in hFOB1.19 cells (Fig. 2C). However, DCAF4 protein levels were not obviously altered in these cells (Fig. 2C). These results suggested that DCAF11 might play a major role because of its stronger interaction with DDB1 compared with DCAF4 in osteosarcoma cells. To confirm this hypothesis, we examined DCAF4 and DCAF11 protein levels in different osteosarcoma cell lines. As shown in Fig. 2D, DCAF11, instead of DCAF4, was significantly induced in U2OS, MG63, Saos-2 and HOS osteosarcoma cell lines compared with hFOB1.19 cells. These results clearly demonstrated that CUL4B forms an E3 complex with RBX11, DDB1 and DCAF11, termed CRL4B^DCAF11^, in osteosarcoma cells, and more importantly, that the components of this complex were highly expressed in osteosarcoma cells.

### CRL4B E3 ligase recognizes p21 as a substrate in human osteosarcoma cells

**Figure 3:**
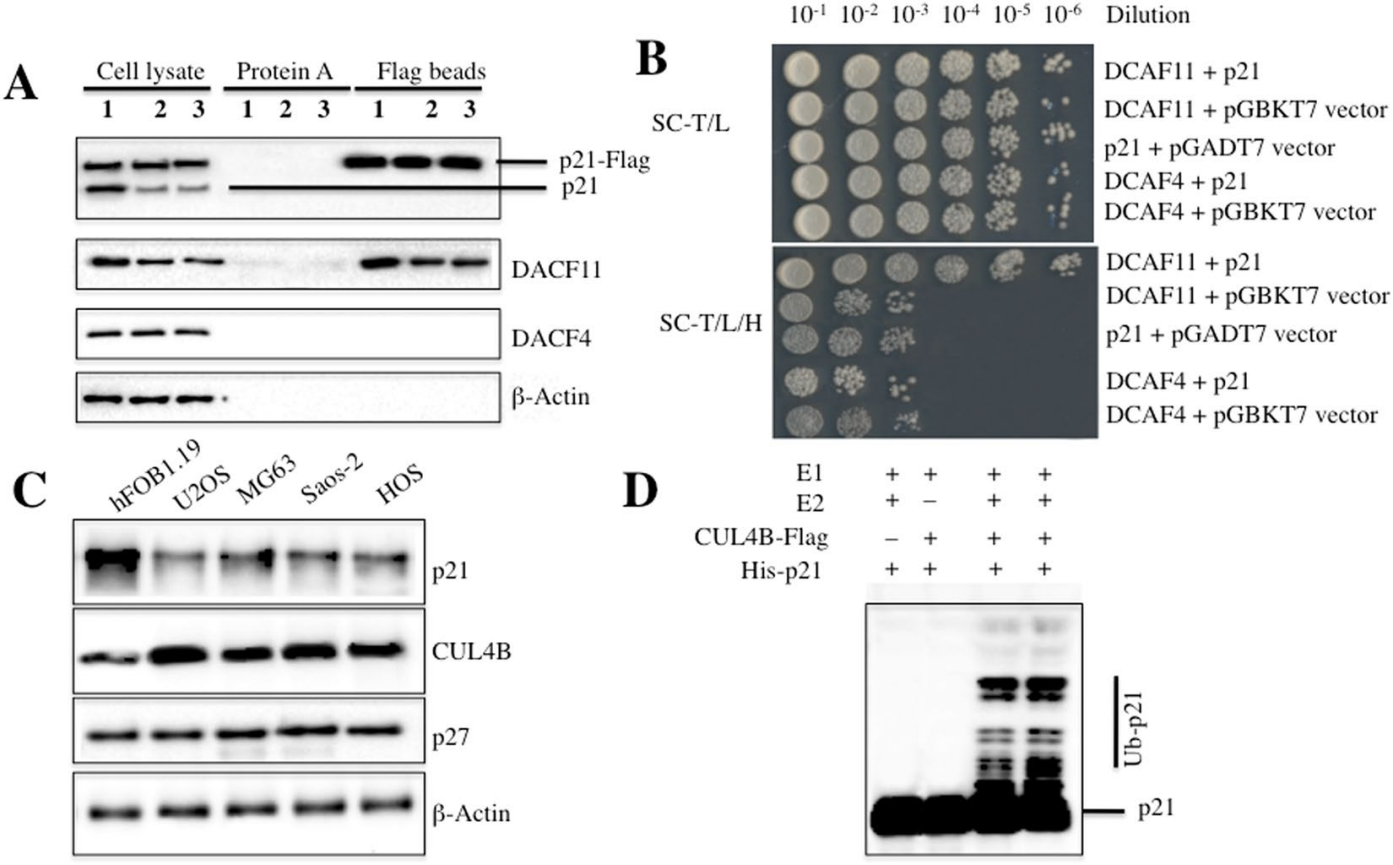
p21 is a target of CRL4B^DCAF11^ E3 ligase in human osteosarcoma cells. (**A**) Flag-tagged p21 associated with DCAF11 *in vivo*. The *pCDNA3-p21-Flag* plasmid was transformed into hFOB1.19 (1), U2OS (2) or Saos-2 (3) cells. After 48 h incubation, Flag-tagged proteins were immunoprecipitated with either protein A agarose or anti-Flag-agarose. The cell lysate and pull-down products were analysed by immunoblotting with anti-p21, anti-DCAF1, anti-DCAF4, anti-DCAF11 or β-Actin antibodies. (**B**) p21 interacted with DCAF11 in yeast. The *pGBKT7-p21* plasmid was co-transformed with *pGADT7-DCAF11* or *pGADT7-DCAF4* into the yeast strain AH109. Growth of the transformed yeast was assayed on media lacking Trp and Leu (top panel) or lacking Trp, Leu, and His (bottom panel). Columns in each panel represent serial 10-fold dilutions. (**C**) p21 was significantly down-regulated in human osteosarcoma cell lines. Cell lysates were applied to WB analyses with specific antibodies against p21, CUL4B, p27, or β-Actin. (**D**) p21 was ubiquitinated by CRL4B^DCAF11^ E3 ligase *in vitro*. The purified His-p21 protein was incubated with E1, with or without E2, and with or without CUL4B-Flag in reaction buffer, followed by immunoblotting for p21.

CRL4 E3 ligases have been reported to recognize many substrates through different DCAFs in different cancer types^12, 13, 15, 16, 18, 27–29^. To identify the substrates of CRL4B^DCAF11^ E3 ligase in osteosarcoma cells, we constructed the *pCDNA3-Flag-HA-DCAF11* vector, a two-epitope tagged vector designed to minimize unspecific binding during purification, which we transfected into hFOB1.19, U2OS and Saos-2 cells. Accordingly, the Flag-HA-DCAF11-associated complex was first immunoprecipitated with anti-Flag agarose, and the Flag epitope was then cleaved off. The resulting supernatant was then immunoprecipitated with anti-HA agarose, followed by elution using an HA peptide. After this two-step purification, HA-DCAF11-containing complexes were enriched and subjected to liquid chromatography tandem-mass spectrometry (LC-MS/MS) analysis. A total of 113 proteins were identified, and we listed the top ten candidates that demonstrated higher MASCOT scores in Supplementary Table 1. One putative DCAF11-interacting protein, p21, exhibited higher tandem MS read values and was previously reported to be a substrate of CRL4A E3 ligase^13^. To verify the interaction between DCAF11 and p21, the *pCDNA3-p21-Flag* vector was constructed and transfected into U2OS cells, p21 was immunoprecipitated with anti-Flag agarose, and DCAF11 was detected using an immunoblot assay. The results revealed that DCAF11, but not DCAF4, was immunoprecipitated by p21 (Fig. 3A). Moreover, we also observed a significant decrease in p21 protein level in U2OS and Saos-2 cell lysates compared with hFOB1.19 lysates (Fig. 3A). To further verify the interaction between p21 and DCAF11, Y2H analyses were also performed using the following combinations of plasmids: *pGADT7−DCAF11* + *pGBKT7−p21*, *pGADT7−DCAF11* + *pGBKT7*, *pGADT7* + *pGBKT7−p21*, *pGADT7−DCAF4* + *pGBKT7−p21*, and *pGADT7−DCAF4* + *pGBKT7*. Accordingly, plasmids were co-transformed into AH109 yeast cells, and the positive colonies were then selected and plated on synthetic dropout medium to determine the interactions between DCAF11 and p21. Our results indicated that DCAF11 interacted with p21, whereas DCAF4 did not (Fig. 3B). These results suggested that p21 might be a substrate of the CRL4B^DCAF11^ E3 ligase.

The fact that components of the CRL4B^DCAF11^ complex were upregulated in osteosarcoma cells suggested that this E3 ligase might be more active and might cause the degradation of its specific substrates. As such, we would expect p21 protein levels to be down-regulated if it was indeed a substrate of CRL4B^DCAF11^ E3 ligase. To verify this hypothesis, we next examined p21 protein levels in different osteosarcoma cell lines. As shown in Fig. 3C, p21 protein levels were significantly lower in the osteosarcoma cell lines compared with hFOB1.19 cells. In addition, we also detected the expression of p27, a target of CRL4A E3 ligase, under the same conditions. No obvious changes were observed in p27 protein levels (Fig. 3C). These results implied that CRL4B^DCAF11^ E3 ligase might directly target p21 for degradation in osteosarcoma cells. To determine whether CRL4B^DCAF11^ E3 ligase ubiquitinates p21 *in vitro*, we reconstituted the ubiquitination reaction using combinations of E1, E2, immunoprecipitated CUL4B-Flag, and purified recombinant His-p21 protein. p21 ubiquitination was detected by immunoblotting for p21. Incubating p21 with E1, E2 and CUL4B-Flag produced species with higher molecular weights than p21, which were absent in reactions lacking E2 or CUL4B-Flag (Fig. 3D), indicating that CRL4B^DCAF11^ E3 ligase can ubiquitinate p21 *in vitro*.

### K16, K154, K161 and K163 are required for p21 ubiquitination

**Figure 4:**
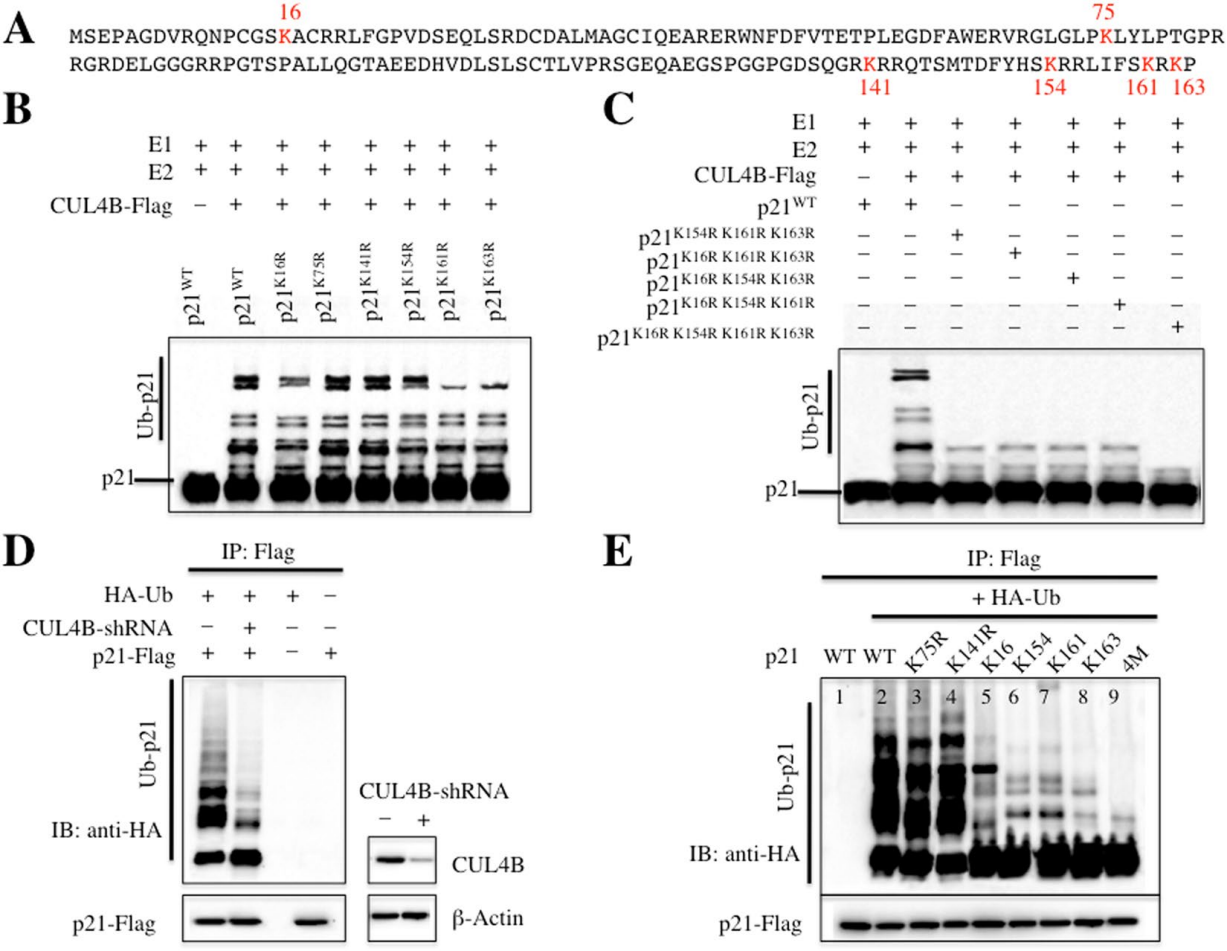
CRL4B^DCAF11^ E3 ligase ubiquitinated p21 at K16, K154, K161 and K163. (**A**) Possible ubiquitination sites of p21. The amino acid sequence of p21 contains six lysines. (**B**,**C**) CRL4B^DCAF11^ E3 ligase ubiquitinated p21 at K16, K154, K161 and K163 *in vitro*. Purified His-p21 and p21 mutants, including p21^K16R^, p21^K75R^, p21^K141R^, p21^K154R^, p21^K161R^, p21^K163R^, p21^K154R K161R K163R^, p21^K16R K161R K163R^, p21^K16R K154R K163R^, p21^K16R K154R K161R^ and p21^K16R K154R K161R K163R^, were incubated with E1, E2, and CUL4B-Flag in ubiquitination reaction buffer, followed by immunoblotting for p21. (**D**) CRL4B^DCAF11^ E3 ligase ubiquitinated p21 *in vivo*. U2OS cells were transfected with p21-Flag and HA-Ubiquitin, followed by retroviral infection of *CUL4B* shRNA. After 48 h, cells were lysed, immunoprecipitated with Flag antibody, and then probed with anti-HA antibody for ubiquitinated p21. Right, demonstrated knockdown of *CUL4B* by shRNA. (**E**) CRL4B^DCAF11^ E3 ligase ubiquitinated p21 at K16, K154, K161 and K163 *in vivo*. U2OS cells were transfected with HA-Ubiquitin and p21-Flag or mutations, including p21^K75R^-Flag, p21^K141R^-Flag, p21^K154R K161R K163R^-Flag (K16), p21^K16R K161R K163R^-Flag (K154), p21^K16R K154R K163R^-Flag (K161), p21^K16R K154R K161R^-Flag (K163) and p21^K16R K154R K161R K163R^-Flag (4 M). After 48 h, cells were lysed, immunoprecipitated with Flag antibody, and then probed with anti-HA antibody for ubiquitinated p21.

The amino acid sequence of p21 contains six lysine (K) residues, K16, K75, K141, K154, K161 and K163 (Fig. 4A). To identify the ubiquitination site(s) of p21, we mutated each of these residues to arginine (R) and evaluated the ubiquitination of these mutants using an *in vitro* ubiquitination assay. As shown in Fig. 4B, a slightly reduced ubiquitination pattern was observed for K16R, K154R, K161R and K163R in reactions with E1, E2 and CUL4B-Flag, but not for K75R and K141R. Furthermore, we created different p21 mutations, including p21^K154R K161R K163R^, p21^K16R K161R K163R^, p21^K16R K154R K163R^, p21^K16R K154R K161R^ and p21^K16R K154R K161R K163R^, to analyse ubiquitination patterns. As shown in Fig. 4C, only faint ubiquitination bands were observed for p21^K154R K161R K163R^, p21^K16R K161R K163R^, p21^K16R K154R K163R^ and p21^K16R K154R K161R^ in reactions with E1, E2 and CUL4B-Flag. Importantly, ubiquitination was completely abolished in the p21^K16R K154R K161R K163R^ mutant (Fig. 4C). Based on the p21 ubiquitination pattern, our results indicated that CRL4B^DCAF11^ E3 ligase catalyses the multi-monoubiquitination rather than the poly-ubiquitination of p21.

To determine whether CRL4B^DCAF11^ E3 ligase was able to ubiquitinate p21 in *vivo*, we first examined p21 ubiquitination with and without suppression of CUL4B by shRNA. As shown in Fig. 4D, inhibiting CUL4B expression significantly reduced p21 ubiquitination, suggesting that CRL4B^DCAF11^ E3 ligase contributed to p21 ubiquitination *in vivo*. To examine whether K16, K154, K161 and K163 were also important for p21 ubiquitination *in vivo*, we first constructed p21^K75R^, p21^K141R^, p21^K154R K161R K163R^ (K16, in which only K16 was present), p21^K16R K161R K163R^ (K154, in which only K154 was present), p21^K16R K154R K163R^ (K161, in which only K161 was present), p21^K16R K154R K161R^ (K163, in which only K163 was present), and p21^K16R K154R K161R K163R^ (4M, in which all four sites were mutated) in the *pCDNA3-Flag* vector and co-transfected them with HA-Ub. Consistent with the *in vitro* results, the *in vivo* ubiquitination analysis also indicated that K16, K154, K161 and K163 were required for p21 ubiquitination, whereas K75 and K141 were not (Fig. 4E). Ubiquitination was almost completely absent in the p21^K16R K154R K161R K163R^ mutant (Fig. 4E).

### Mutating K16R, K154R, K161R and K163R enhances p21 stability and decreases osteosarcoma cell proliferation

**Figure 5:**
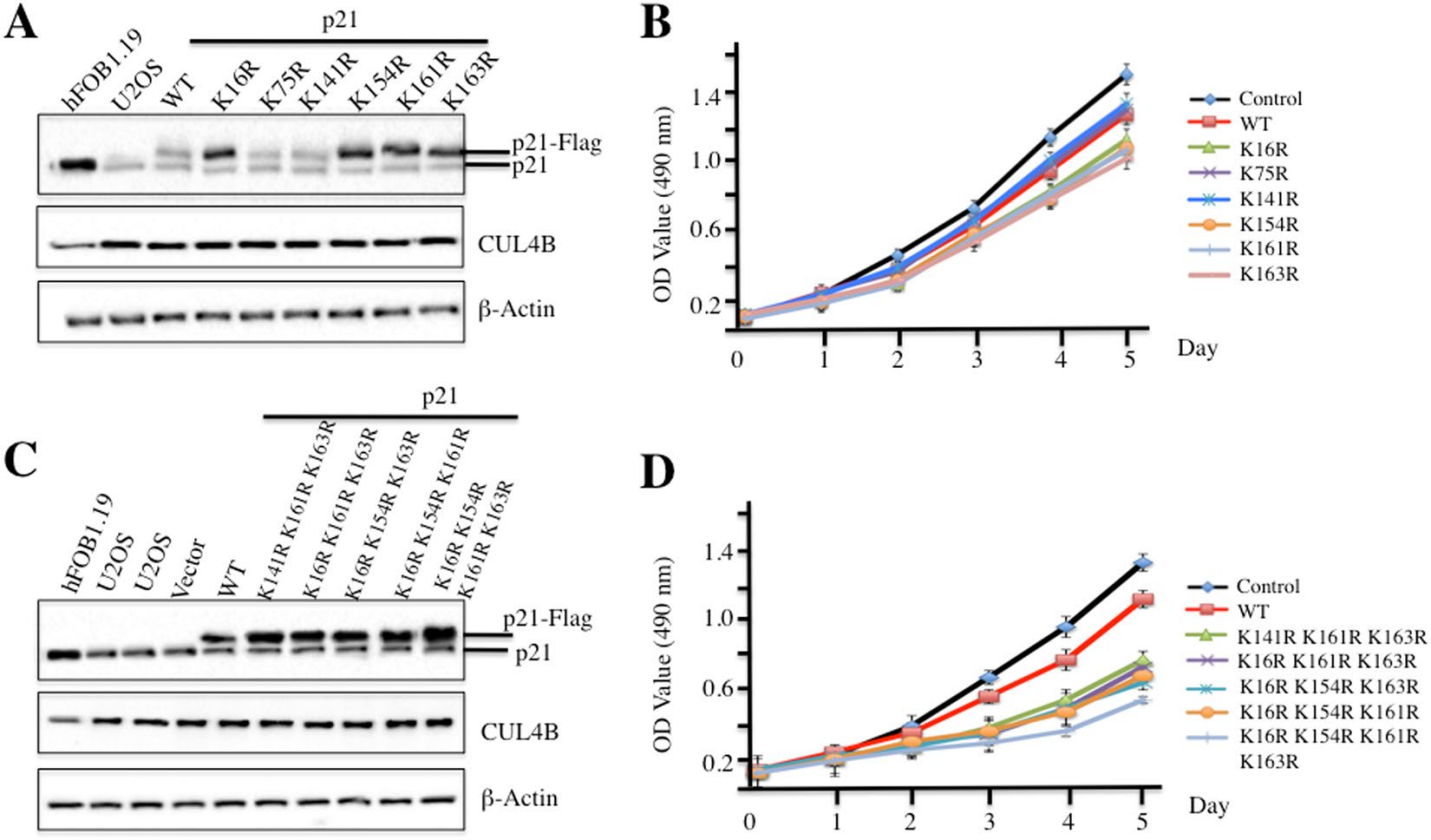
p21 stability affects osteosarcoma cell proliferation. (**A–C**) Mutations of ubiquitination sites resulted in p21 accumulation *in vivo*. U2OS cells were transfected with p21-Flag or p21 mutants, including p21^K16R^-Flag, p21^K75R^-Flag, p21^K141R^-Flag, p21^K154R^-Flag, p21^K61R^-Flag, p21^K163R^-Flag, p21^K154R K161R K163R^-Flag, p21^K16R K161R K163R^-Flag, p21^K16R K154R K163R^-Flag, p21^K16R K154R K161R^-Flag, and p21^K16R K154R K161R K163R^-Flag. After 48 h incubation, cells were lysed and immunoprecipitated with anti-p21, anti-CUL4B or β-Actin antibody. (**B–D**) Mutations of ubiquitination sites decreased cell proliferation. Cells used in (**A** and **C**) were applied to MTT assays to evaluate cell proliferation, and cell viability was determined at 490 nm.

The *in vitro* and *in vivo* results revealed that K16, K154, K161 and K163 were required for p21 ubiquitination. To determine whether mutating these four sites affected cell proliferation, we first constructed p21^K16R^, p21^K75R^, p21^K141R^, p21^K154R^, p21^K161R^, p21^K163R^, p21^K154R K161R K163R^, p21^K16R K161R K163R^, p21^K16R K154R K163R^, p21^K16R K154R K161R^ and p21^K16R K154R K161R K163R^ in the *pCDNA3-Flag* vector and then transfected each individual plasmid into U2OS cells. After 48 h, the levels of these mutated proteins were determined, and the results showed that the p21^K16R^-Flag, p21^K54R^-Flag, p21^K161R^-Flag and p21^K163R^-Flag proteins were much more stable than the p21^K75R^-Flag and p21^K141R^-Flag proteins (Fig. 5A), which further suggested that mutating K16R, K154R, K161R or K163R might attenuate ubiquitination levels, thereby causing the accumulation of these proteins. Next, cell proliferation rates in U2OS cells expressing these single-mutated proteins were also determined using an MTT kit. Interestingly, cells harbouring K16R, K154R, K161R or K163R mutations showed slight reductions in cell proliferation compared with cells transfected with p21^WT^-Flag, p21^K75R^-Flag or p21^K141R^-Flag (Fig. 5B). In addition, we also determined the stability of other triple and quadruple mutants, and p21^K154R K161R K163R^-Flag, p21^K16R K161R K163R^-Flag, p21^K16R K154R K163R^-Flag, p21^K16R K154R K161R^ -Flag and p21^K16R K154R K161R K163R^-Flag were much more stable than p21^WT^ (Fig. 4C). Of these triple and quadruple mutants, the quadruple mutant showed the highest abundance (Fig. 4C). Consistent with these results, cells transfected with these mutants exhibited significantly reduced proliferation rates compared with cells transfected with p21^WT^ (Fig. 4D), suggesting the importance of K16, K154, K161 and K163 in maintaining cell proliferation.

Because mutating these four critical sites increased p21 stability, we next sought to determine whether these mutants had longer half-lives. Accordingly, we transfected p21^WT^-Flag, p21^K16R^-Flag, p21^K75R^-Flag, and p21^K16R K154R K161R K163R^-Flag into U2OS cells. To ensure that the cells expressed similar levels of Flag-tagged p21, we treated U2OS cells immediately after transfection with the proteasome inhibitor MG132 and by changing the medium every 12 h. After 48 h, cells were incubated in fresh medium without MG132, and samples were collected every 20 min. Then, Flag-tagged p21 were detected by immunoblotting, which indicated that the half-lives of p21^WT^-Flag and p21^K75R^-Flag were approximately 80 min (Supplementary Figure 2), whereas the half-life of p21^K16R^-Flag was nearly 100 min (Supplementary Figure 2). Interestingly, p21^K16R K154R K161R K163R^-Flag was very stable, and we did not observe obvious degradation even at the 100-min time point (Supplementary Figure 2). These results further suggested that the K16R, K154R, K161R and K163R sites are important for p21 ubiquitination.

### Knocking down components of the CRL4B^DCAF11^ complex inhibits osteosarcoma cell proliferation, induces S phase cell cycle arrest, and decreases colony formation rates

**Figure 6:**
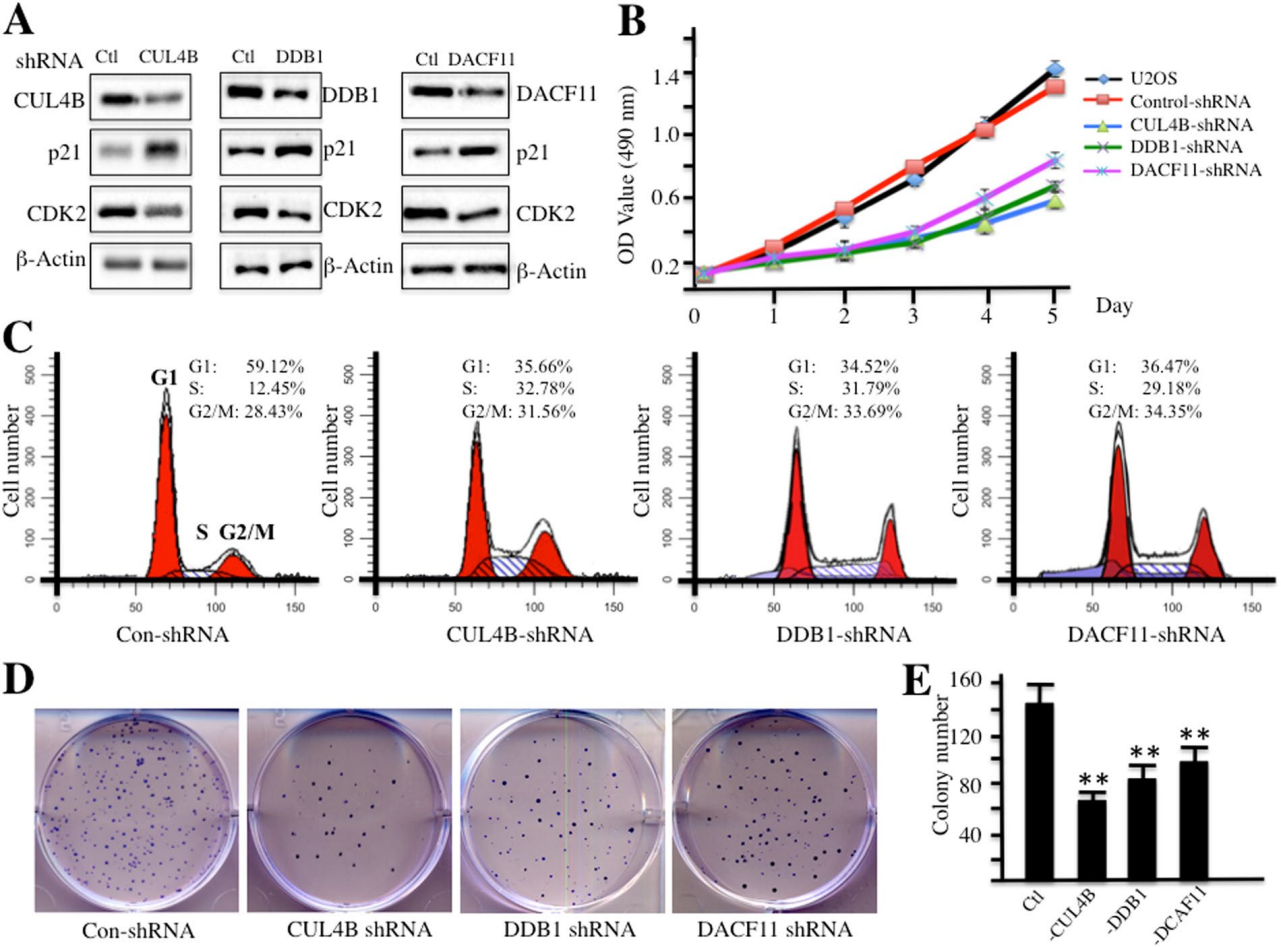
Knocking down CRL4B^DCAF11^ complex components inhibits osteosarcoma cell growth. (**A**) Knocking down CRL4B^DCAF11^ complex components leads to the accumulation of p21. U2OS cells transfected with Con-shRNA, CUL4B-shRNA, DDB1-shRNA, or DCAF11-shRNA. After 48 h incubation, cells were lysed and probed with anti-CUL4B, DDB1, DCAF11, p21, CDK2, or β-Actin antibody. (**B**) Knocking down CRL4B^DCAF11^ complex components inhibits osteosarcoma cell proliferation. Cells used in A were applied to the MTT assay to evaluate cell proliferation, and cell viability was determined at 490 nm. (**C**) Knocking down CRL4B^DCAF11^ complex components induces cell cycle arrest at S phase. Cells used in A were subjected to flow cytometry analysis of cell cycle distribution. (**D,E**) Knocking down CRL4B^DCAF11^ complex components decreased colony formation rates. Cells (1 × 10^3^) used in A were seeded onto 12-well plates, and cells were cultured with 0.1 ml of fresh medium containing 0.5% FBS for two weeks. Then, cells were stained with 0.5% crystal violet (**D**), and colony numbers were counted by a gel documentation system (**E**).

To investigate whether the components of the CRL4B^DCAF11^ complex, including CUL4B, DDB1 and DCAF11, were able to regulate p21 protein levels, we used shRNAs to knock down the expression of these three genes. As shown in Fig. 6A, shRNA-transfected cells exhibited significant reductions in the levels of the target proteins and obvious increases in p21 levels. Given that p21 is a well-known inhibitor of CDK2, a kinase required for the transition from G1 to S phase, we next sought to determine whether CDK2 protein levels were down-regulated following the induction of p21 in cells with CUL4B, DDB1 or DCAF11 knock down. As expected, we observed a significant reduction in CDK2 (Fig. 6A). In addition, cell proliferation assays were also performed to evaluate the down-regulation of components of the CRL4B^DCAF11^ E3 ligase, and as shown in Fig. 6B, knocking down CUL4B, DDB1 or DCAF11 inhibited cell proliferation.

Given that the down-regulation of CRL4B^DCAF11^ components led to the accumulation of p21 and to the reduction of CDK2, two key regulators of cell cycle progression, we proposed that the down-regulation of these three members should affect cell cycle progression. To verify this hypothesis, we subjected cells with reduced CUL4B, DDB1 or DCAF11 expression to flow cytometry analyses. As expected, osteosarcoma cells expressing lower levels of CUL4B, DDB1 or DCAF11 exhibited dramatically higher percentages of cells in S phase (Fig. 6C). These results suggested that S phase cell cycle arrest might contribute to the cell proliferation inhibition observed upon the down-regulation of CRL4B^DCAF11^ E3 ligase components. In addition, colony formation rates significantly decreased after the down-regulation of CRL4B^DCAF11^ E3 ligase components (Fig. 6D and E).

## Discussion

**Figure 7:**
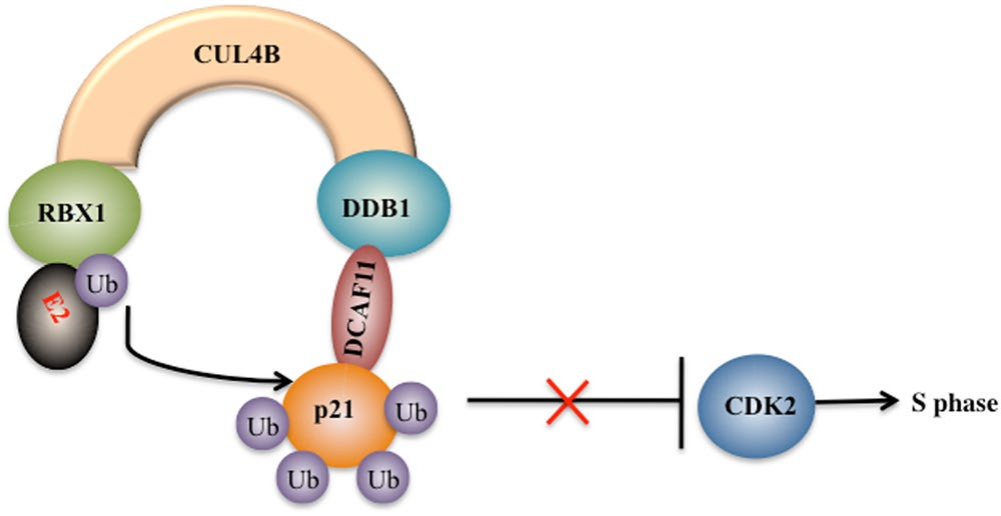
Schematic diagram of the CRL4B^DCAF11^ ubiquitin complex in human osteosarcoma cells. CUL4B serves as a scaffold that binds to adaptor protein DDB1 and the substrate receptor protein DCAF11 at the N terminus and interacts with RING protein RBX1 at the C terminus, thereby forming the CRL4B E3 ligase. The CRL4B E3 ligase can promote ubiquitin transfer from RBX1-bound E2 to the specific substrate p21 in osteosarcoma cells, leading to p21 ubiquitination. The degradation of p21 diminishes the inhibition of CDK2, thereby resulting in cell cycle arrest at S phase.

Accumulating evidence suggests critical roles for Cullin family proteins and their associated CRL E3 ligases in tumourigenesis^12, 13, 15–17, 27–29^. In the present study, we discovered that CUL4B interacted with DDB1, RBX1 and DCAF11 to form an E3 ligase known as CRL4B^DCAF11^. We also determined that CRL4B^DCAF11^ directly targeted p21 for multi-monoubiquitination at K16, K154, K161 and K163 through *in vitro* and *in vivo* studies. Moreover, knocking down any component of the CRL4B^DCAF11^ complex by shRNA resulted in p21 accumulation, cell growth inhibition, S phase cell cycle arrest, and reduced colony formation rates. Our results provide evidence for a potential new DCAF11 pathway in which DCAF11 associates with CUL4B and DDB1 to target p21 for proteolysis in human osteosarcoma cells (Fig. 7).

CUL4A and CUL4B, two paralogs in mammalian cells, share high amino acid sequence similarity and have partially redundant functions in many biological processes, such as nucleotide excision repair (NER)^31–33^. Our previous studies have indicated that CUL4B is upregulated in human osteosarcoma cells^26^. However, we did not examine the expression of other Cullin members to determine if only CUL4B was specifically upregulated. As such, in the present study, we first detected the expression of all seven Cullin members in different human osteosarcoma cell lines. CUL4B mRNA and protein were both specifically overexpressed in these human osteosarcoma cell lines, although CUL4A also exhibited a slight induction under the same conditions. Thus, CUL4B was considered to be specifically overexpressed in human osteosarcoma cells, possibly through an unknown transcriptional mechanism, which also indicated that CUL4B has a unique role in human osteosarcoma cells.

In addition, CUL4B has been demonstrated to interact with DDB1 and RBX1 in many studies^12, 34^. Similarly, we also examined and confirmed these interactions through *in vivo* Co-IP and in *vitro* Y2H studies. The RBX1-CUL4B-DDB1 complex interacts with a number of substrate receptors known as DCAFs (e.g., DDB2, CSA, COP1 and DCAF1–19) to recognize thousands of substrates^12, 34^. One limitation of our studies is that we did not screen for DCAFs that associate with CUL4B in osteosarcoma cells; instead, we only examined the expression of DCAF1, 4, 8, 11 and 15, and not the expression of other DCAFs. Fortunately, we found that DCAF11 had a similar expression pattern to CUL4B and was upregulated in different osteosarcoma cells. To determine whether DCAF11 was the only DCAF protein that interacted with DDB1 in osteosarcoma cells, it is necessary to utilize DDB1 as the bait to screen associating proteins and then select candidate DCAFs to examine expression in osteosarcoma cells.

We screened proteins that interacted with DCAF11 and identified a target of CRL4B^DCAF11^ E3 ligase known as p21. p21 protein levels were significantly decreased in osteosarcoma cells. Surprisingly, although p21 and p27 function in the regulation of cell cycle progression, we observed no changes in p27 levels in osteosarcoma cells. A number of studies have shown that p21 can be ubiquitinated by several E3 ligases, such as CRL4^Cdt2^ and SCF^Skp2^ ^11–14^. Human CRL4^Cdt2^ targets and ubiquitinates p21 during S phase in a proliferating cell nuclear antigen (PCNA)-dependent manner^13^. Inactivation of CRL4^Cdt2^ E3 ligase prevents the nuclear export of CDC6 during S phase, and this block of nuclear export depends on p21^35^. SCF^Skp2^ E3 ligase participates in the degradation of p21 in S phase. Knocking out Skp2 in mouse embryo fibroblasts results in higher p21 levels than in wild-type fibroblasts in S phase because of the deficient p21 ubiquitination^36^. Our *in vivo* and *in vitro* ubiquitination studies indicated that CRL4B^DCAF11^ E3 ligase was able to ubiquitinate p21 at four different sites and failed to ubiquitinate the p21^K16R K154R K161R K163R^ mutant *in vitro* and *in vivo*. Interestingly, cells expressing p21^K16R^, p21^K154R^, p21^K161R^, p21^K163R^, p21^K154R K161R K163R^, p21^K16R K161R K163R^, p21^K16R K154R K163R^, p21^K16R K154R K161R^ or p21^K16R K154R K161R K163R^ exhibited lower cell proliferation rates. Our results strongly indicate that p21 multi-monoubiquitination is important for maintaining p21 function. By comparing the ubiquitination pattern of p21 with previous publications, we found a major difference: some studies found that even when all lysines in p21 were mutated, p21 was still ubiquitinated at the N terminus^37–39^. In our case, only K16, K154, K161 and K163 were required for p21 ubiquitination, and the quadruple mutant lacking all four of these sites could not be ubiquitinated. One possible explanation for these different ubiquitination patterns is that p21 can be modified by different E3 ligases in different cell types and that different E3 ligases might recognize different lysines. In our study, we did not detect ubiquitination at the p21 N terminus because we detected no p21 ubiquitination after mutating K16, K154, K161 and K163. Thus, it was not necessary to further detect ubiquitination at the p21 N terminus. On the other hand, these different ubiquitination patterns provided a new view on the modification of p21. In addition, in cells depleted of CUL4B, DDB1 or DCAF11, p21 degradation was impaired, which resulted in cell cycle arrest at S phase, cell growth inhibition and a reduced colony formation rate. This provides a strategy to target different components of this E3 ligase in osteosarcoma therapy.

In our LC-MS/MS results (Supplementary Table 1), we found that CDK2 was pulled down by CUL4B. We also observed a significant decrease in CDK2 levels after down-regulation of CUL4B, DDB1 or DCAF11 in osteosarcoma cells (Fig. 6A), which raised the question of whether DCAF11 is responsible for the degradation of CDK2. To answer this question, we performed *in vivo* Co-IP and *in vitro* Y2H assays to determine whether DCAF11 interacts with CDK2. Unfortunately, we did not detect an interaction between these two proteins (Supplementary Figure 3). Thus, CDK2 might be only a CUL4B-associated protein, and its down-regulation in osteosarcoma cells expressing lower CUL4B, DDB1 or DCAF11 might not be regulated by DCAF11 but might rather be regulated by p21 induction, as p21 is an inhibitor of CDK2.

In summary, our results demonstrate that CUL4B forms an E3 ligase with RBX1, DDB1 and DCAF11 to recognize p21 as a substrate in human osteosarcoma cells. Our study offers insights into how CUL4B specifically functions in osteosarcoma cells and demonstrates that osteosarcoma cells have a unique CRL4B^DCAF11^ E3 ligase system that functions in the regulation of cell cycle progression.

## Materials and Methods

### Cell lines and growth conditions

The hFOB1.19 osteoblast cell line and the four osteosarcoma cell lines U2OS, MG63, HOS and Saos-2 were purchased from the American Type Culture Collection (ATCC, USA) and maintained in Dulbecco’s modified Eagle’s medium (DMEM) (Gibco, USA) supplemented with 10% heat-inactivated foetal bovine serum (FBS) (Gibco, USA) and penicillin-streptomycin (100 U/ml each). All cell lines were cultured in an incubator at 37 °C with 95% air and 5% CO_2_. Cells were split twice per week when they were approximately 80% confluent.

### Western blotting

Total cell extracts were prepared using Pierce IP lysis buffer (Thermo Fisher Scientific, USA) according to the manufacturer’s instructions. Equal amounts of proteins were subjected to WB analysis. The antibodies used for WBs were anti-β-Actin (mouse, Sigma, USA), anti-CUL1 (rabbit, Thermo Fisher Scientific, USA), anti-CUL2 (mouse, Santa Cruz Biotechnology, USA), anti-CUL3 (rabbit, Cell Signaling Technology, USA), anti-CUL4A (mouse, Sigma, USA), anti-CUL4B (mouse, Sigma, USA), anti-CUL5 (rabbit, Abcam, USA), anti-CUL7 (rabbit, OriGene, USA), anti-Flag (mouse, Sigma, USA), anti-RBX1 (rabbit, Cell Signaling Technology, USA), anti-DCAF1 (rabbit, Abcam, USA), anti-DCAF4 (rabbit, Sigma, USA), anti-DCAF8 (rabbit, Sigma, USA), anti-DCAF11 (rabbit, Sigma, USA), anti-DCAF15 (rabbit, Sigma, USA), anti-DDB1 (rabbit, Sigma, USA), anti-p21 (rabbit, Sigma, USA), and anti-p27 (mouse, Abcam, USA). Signals from WBs were recorded using a ChemiDoc MP (Bio-Rad, USA). All experiments were performed in triplicate.

### Real-time PCR analysis

Total RNA was isolated using TRIzol reagent (Invitrogen, USA), and cDNA was synthesized using the SuperScript First-Strand Kit (Invitrogen, USA) after the removal of genomic DNA using RNase-free DNase I (Takara, Japan). The resulting cDNAs were applied to quantitative RT-PCR analysis using the specific primers listed in Supplementary Table 2. The PCR procedure was carried out as follows: 95 °C for 1 min, followed by 45 cycles of 95 °C for 10 sec and 68 °C for 30 sec, and then 4 °C for 10 min. All experiments were performed in triplicate, and gene expression was assessed using the 2^−ΔCt^ method by normalizing to β-Actin, the internal control, as previously described^40^.

### Immunoprecipitation and mass spectrometry analysis

Cells transfected with *pCDNA3-Flag-HA-DCAF11* or *pCDNA3-Flag-HA* were lysed with Pierce IP lysis buffer (Thermo Fisher Scientific, USA) according to the manufacturer’s instructions and supplemented with 1x protease inhibitors (Roche, USA). Lysates were subjected to IP analysis using anti-Flag-agarose (Sigma, USA). Briefly, lysates were incubated with beads for 4 h at 4 °C. Then, beads were washed five times with lysis buffer, and proteins were eluted with Flag peptide (Sigma, USA) for 2 h at room temperature. HA-tagged DCAF11 protein was visualized on an SDS gel and stained with Coomassie Brilliant Blue R 250 as previously described^41^. The stained protein bands corresponding to the DCAF11 protein were digested with trypsin and analysed by LC-MS/MS. The MS/MS spectra were searched against the NCBI database using a local MASCOT search engine (V.2.3).

### Yeast two-hybrid assay

To determine the interactions between CUL4B and DDB1, CUL4B and RBX1, DCAF11 and p21, and DCAF4 and p21 in yeast cells, the coding sequence of either CUL4B or p21 was cloned into the *pGBKT7* vector between the *Nde*I and the *BamH*I sites. The coding sequence of DDB1, RBX1, DCAF4 or DCAF11 was cloned into the *pGADT7* vector between the *Eco*RI and the *Bam*HI sites. The following combinations of plasmids, including *pGBKT7−CUL4B* + *pGADT7−DDB1*, *pGBKT7−CUL4B* + *pGADT7*, *pGADT7-DDB1* + *pGBKT7*, *pGBKT7−CUL4B* + *pGADT7−RBX1*, *pGADT7−RBX1* + *pGBKT7*, *pGBKT7−p21* + *pGADT7−DCAF11*, *pGBKT7−DCAF11* + *pGBKT7*, *pGBKT7−p21* + *pGADT7*, *pGBKT7−p21* + *pGADT7−DCAF4*, and *pGBKT7−DCAF4* + *pGBKT7*, were co-transformed into AH109 yeast cells. The transformed yeast cells were selected on synthetic complete medium without Trp and Leu (SC-T/L). Interactions were determined on synthetic complete medium lacking Trp, Leu, and His (SC-T/L/H).

### Plasmid and shRNA transfection

For plasmid transfection, cells were transfected using Lipofectamine 2000 (Invitrogen, USA) in suspension with 100 ng of plasmid. Cells were immediately transferred to 0.5 ml of DMEM medium with FBS in 24-well plates and incubated at 37 °C for 48 h.

Specific knockdown of *CUL4B, DDB1* and *DCAF1* expression in U2OS cells was carried out with a lentiviral system. The shRNAs, including *shCUL4B* (TRCN0000006532), *shDDB1* (TRCN0000082856), and *shDCAF11* (TRCN0000138902), were purchased from Sigma (USA). Briefly, the lentiviruses containing either negative control shRNA or *shCUL4B*, *shDDB1* and *shDCAF11* were transfected into U2OS cells following standard procedures. After transfection for 24 h, the virus-infected cells were selected with puromycin (1 μg/ml) for 48 h and then used for the subsequent experiments.

### Cell cycle analysis by flow cytometry

Cells that were approximately 80% confluent were washed twice with ice-cold 1x PBS and then treated with 0.25% trypsin-EDTA. The cell suspension was fixed with 70% ethanol at 4 °C for 24 h. Cells were then washed with 1% bovine serum albumin (BSA) in 1x PBS and then stained for total DNA content with a solution containing 50 μg/mL propidium iodide (PI), 50 μg/mL RNase, 0.1% Triton X-100, and 0.1 mM EDTA in PBS for 30 min at 37 °C. Cell cycle distribution was analysed by flow cytometry (BD Biosciences, USA) and analysed with CellQuest software (BD Biosciences).

### Cell proliferation and colony formation assays

Cell proliferation assays were performed as previously described^42^. Briefly, cells were transfected with different p21 plasmids, or *shCUL4B*, *shDDB1* or *shDCAF11* were plated onto 96-well plates for 0 h, 24 h, 48 h, 72 h, 96 h or 120 h. Then, a cell proliferation kit (Thermo Fisher Scientific, USA) was used to determine cell viability at 490 nm according to the manufacturer’s instructions.

For colony formation assays, cells at approximately 80% confluence were diluted and seeded at approximately 1000 cells per well. After a 12-h incubation in a 37 °C humidified atmosphere containing 5% CO_2_, cells were transfected with *shCUL4B*, *shDDB1* or *shDCAF11* and then continuously cultured with DMEM medium for 14 days; the medium was changed every 3 days. Cell colonies were stained for 15 min with a solution containing 0.5% crystal violet and 25% methanol, followed by three rinses with ddH_2_O to remove excess dye. The colony numbers were counted by a gel documentation system (Bio-Rad Laboratories, Inc.).

### *In vitro* and *in vivo* ubiquitination analysis

For the *in vitro* p21 ubiquitination assay, immunoprecipitated CUL4B-Flag was sequentially washed with lysis buffer (20 mM Tris-HCl, pH 8.0, 0.5% NP-40, 1 mM EDTA, and 150 mM NaCl), LiCl buffer (50 mM Tris-HCl, pH 8.0, and 0.5 M LiCl), and ubiquitination buffer (25 mM Tris-HCl, pH 7.5, 5 mM MgCl_2_, and 2 mM NaF) and then eluted with Flag peptide in ubiquitination buffer. The pET28a-His-p21 vector was transformed into *Escherichia coli* strain BL21 (DE3), and the recombinant His-tagged p21 protein was purified with Ni-NTA magnetic agarose beads (Clontech, USA) according to the manufacturer’s protocol. Ubiquitination reactions were carried out with His-p21 (substrate), CRL4B complex, 0.1 μM E1 (Sigma, USA), 0.4 μM E2 (Sigma, USA), and 10 μg of bovine ubiquitin (Sigma, USA) in ubiquitination buffer supplemented with 2 mM ATP and 0.6 mM DTT at 37 °C for 75 min, and reactions were terminated by boiling in 2x SDS-PAGE loading buffer. Ubiquitination was analysed by immunoblotting with the anti-p21 antibody.

For the *in vivo* ubiquitination assay, Flag-tagged p21 or p21 mutants were co-expressed in U2OS cells with HA-ubiquitin for 48 h. Lysates were prepared by boiling cells with buffer containing 2% SDS, 150 mM NaCl, and 10 mM Tris-HCl, pH 8.0, and then diluted 10-fold with buffer composed of 10 mM Tris-HCl, pH 8.0, 150 mM NaCl, 0.1% NP-40, 1 mM DTT, 2 mM EDTA, and 1x protease inhibitor (cocktail). Cell lysates were incubated with Flag beads for 2 h at 4 °C with rotation, and beads were washed five times with buffer containing 10 mM Tris-HCl, pH 8.0, 1 M NaCl, 1 mM EDTA, and 1% NP-40. The resin was boiled in 2x SDS-PAGE loading buffer, and ubiquitination was analysed by immunoblotting with an anti-HA antibody.

### Statistical analysis

All experiments were independently replicated at least three times. Statistical analyses were performed using two-sided Student’s *t* tests. Significance was set at *P* < 0.05.

## Electronic supplementary material

Supplementary figures and tables
