# Supplementary material for: CRL4BDCAF11 E3 ligase targets p21 for degradation to control cell cycle progression in human osteosarcoma cells
Source: Sci Rep. 2017 Apr 26;7:1175. doi: 10.1038/s41598-017-01344-9 (PMC5430835; doi:10.1038/s41598-017-01344-9)
Supplement: Supplementary file 1 — Supplementary figures and tables [file 41598_2017_1344_MOESM1_ESM.pdf]

CRL4B^{DCAF11} E3 ligase targets p21 for degradation to control cell cycle progression in human osteosarcoma cells

Zhi Chen[1,#], Kun Wang[1,#], Canglong Hou[2,#], Kaibiao Jiang[1], Bin Chen[1], Jianwei Chen[1], Lifeng Lao[1], Lie Qian[1], Guibin Zhong[1], Zude Liu[1], Caiguo Zhang[3,*], Hongxing Shen[1,*]

[1]Department of Spine Surgery, Renji Hospital, School of Medicine, Shanghai Jiao Tong University, Shanghai, China

[2]Department of Orthopedics, Changhai Hospital, Secondary Military Medical University, Shanghai, China

[33]Department of Dermatology, University of Colorado Anschutz Medical Campus, Aurora, CO, USA

[#] These authors contribute equally to this work.

* Corresponding: caiguo.zhang@ucdenver.edu and shenhxgk@126.com

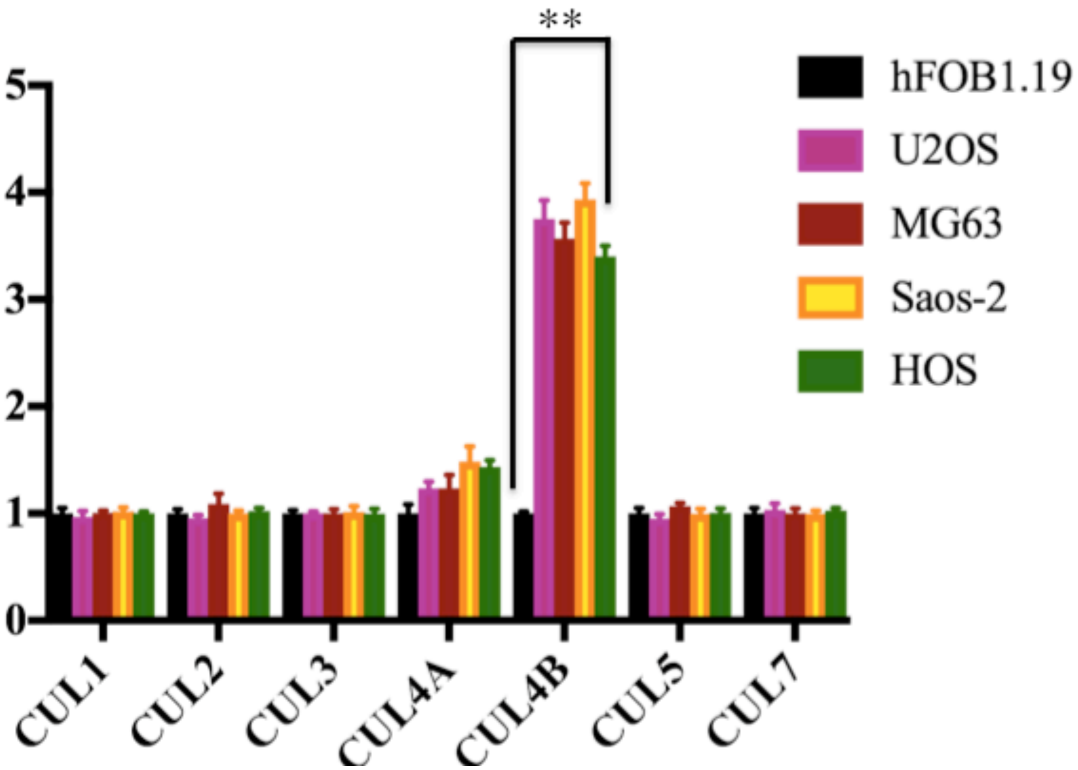

Supplementary Figure 1. CUL4B is upregulated in human osteosarcoma cells.

Protein levels in Figure 1B were normalized against β−Actin. ***P*<0.001.

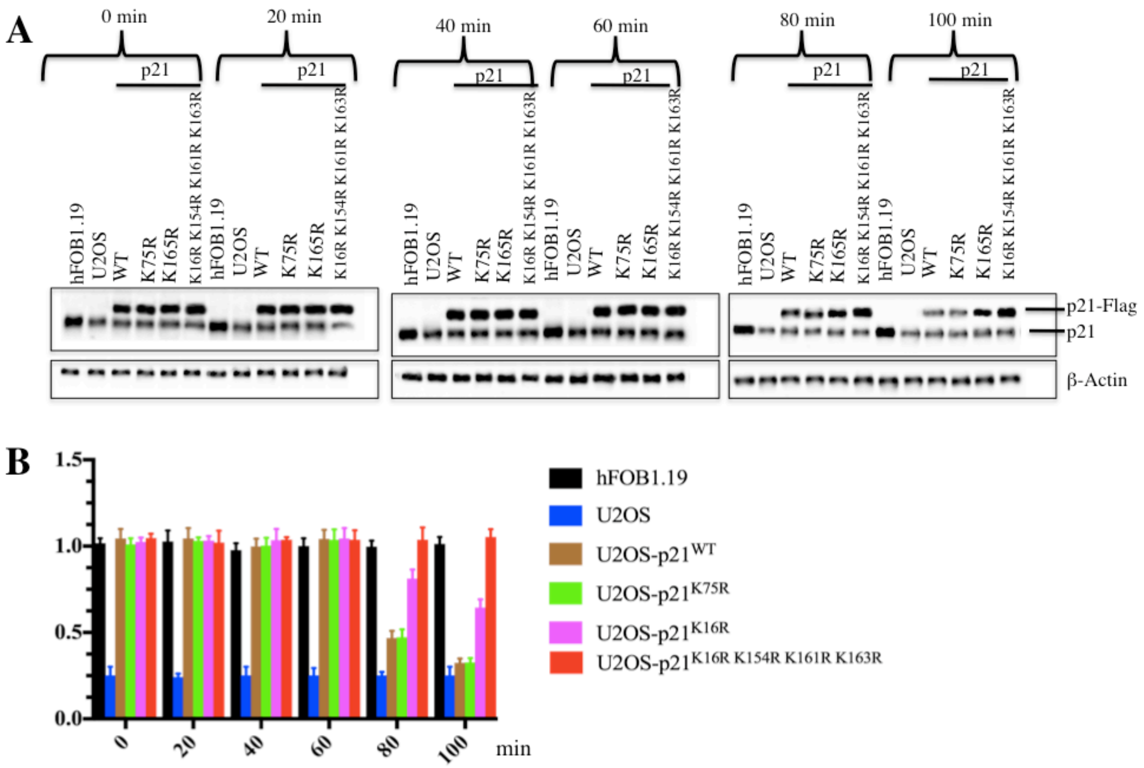

Supplementary Figure 2. The ubiquitination site mutants have longer half-lives.

(A) The p21$^{\text{K16R K154R K161R K163R}}$-Flag protein has a longer half-life. The p21^{WT}-Flag, p21$^{\text{K16R}}$-Flag, p21$^{\text{K75R}}$-Flag, or p21$^{\text{K16R K154R K161R K163R}}$-Flag plasmid was transfected into U2OS cells, followed by immediate treatment with MG132 after transfection and by changing the medium every 12 h. After 48 h, cells were incubated with fresh medium without MG132, and samples were collected every 20 min. The cell lysates were subjected to WB to determine Flag tagged proteins by using anti-p21 antibody. β-Actin was used as a loading control. The half-lives were calculated from three independent experiments and are presented as the means ± SE. **(B)** Normalization of proteins levels in (A).

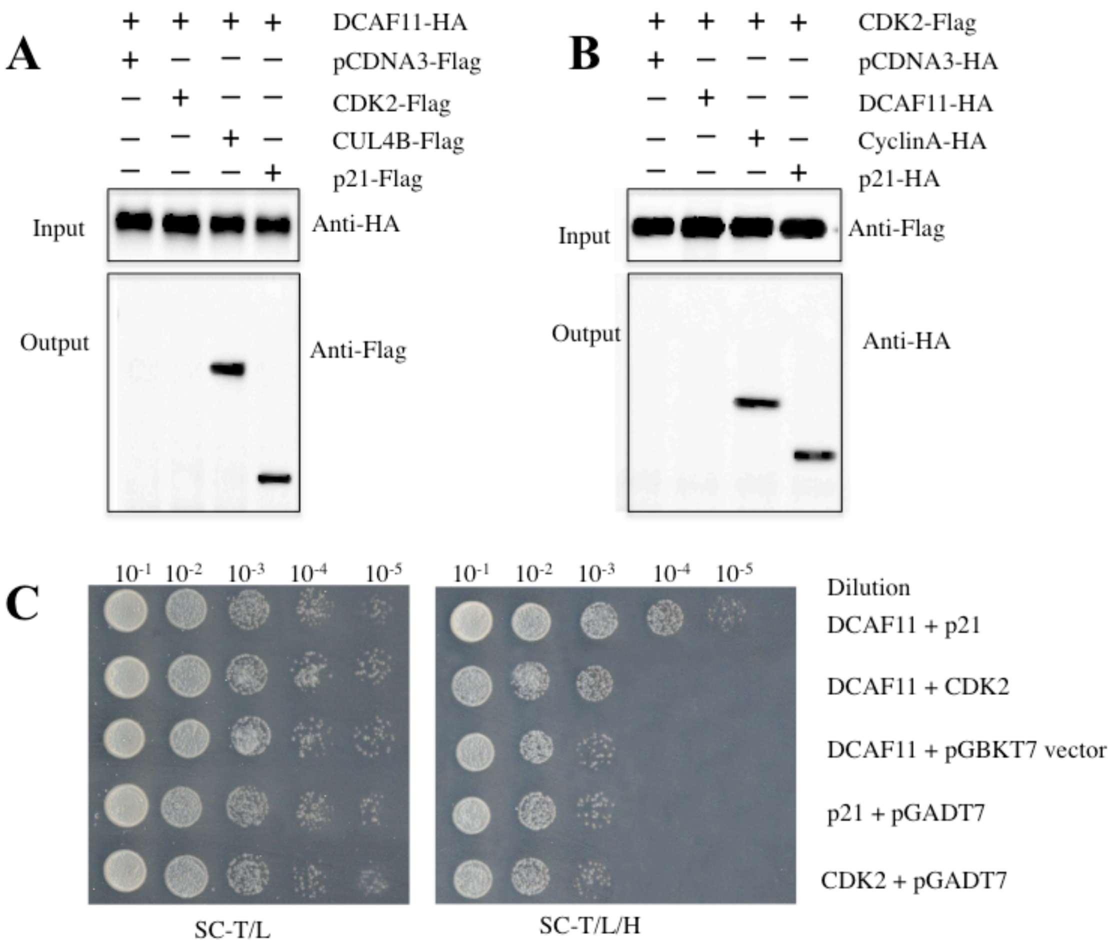

Supplementary Figure 3. DCAF11 does not interact with CDK2.

(A–B) DCAF11 does not interact with CDK2. U2OS cells co-transfected with *pCDNA3-DCAF11-HA* + *pCDNA3-Flag, pCDNA3-DCAF11-HA* + *pCDNA3-CDK2-Flag*, *pCDNA3-DCAF11-HA* + *pCDNA3-CUL4B-Flag*, *pCDNA3-DCAF11-HA* + *pCDNA3-p21-Flag*, *pCDNA3-CDK2-Flag* + *pCDNA3-HA*, *pCDNA3-CDK2-Flag* + *pCDNA3-DCAF11-HA*, *pCDNA3-CDK2-Flag* + *pCDNA3-CyclinA-HA*, or *pCDNA3-CDK2-Flag* + *pCDNA3-p21-HA* were subjected to Co-IP analysis with anti-HA agarose **(A)** or anti-Flag-agarose **(B)**. The pull-down products were then analysed by immunoblotting with anti-Flag and anti-HA antibodies. **(C)** DCAF11 does not interact with CDK2 in yeast. *pGADT7-DCAF11* + *pGBKT7-p21*, *pGADT7-DCAF11* + *pGBKT7-CDK2*, *pGADT7-DCAF11* + *pGBKT7*, *pGBKT7-p21* + *pGADT7*, or *pGBKT7-CDK2* + *pGADT7* were co-transformed into the yeast strain AH109, followed

by plating onto synthetic dropout medium (lacking Trp and Leu, SC-T/L). Positive colonies were then used to determine interactions on medium lacking Trp, Leu, and His (SC-T/L/H). Serial 10-fold dilutions are indicated.

Supplementary Table-1. Main candidate proteins interacting with DCAF11 in human osteosarcoma cells

Accession Number	Protein ID	Symbol	Molecular Weight	MASCOT Score
Q16531	Damaged DNA binding protein 1	DDB1	127 kDa	974
Q92905	COP9 Signalosome Subunit 5	COPS5	37.6 kDa	903
Q13620	Cullin 4B	CUL4B	104 kDa	873
O95831	Apoptosis Inducing Factor, Mitochondrion-Associated 1	AIFM1	67 kDa	661
Q7l5N1	COP9 Signalosome Subunit 6	COPS6	36 kDa	509
Q14010	Cyclin-Dependent Kinase Inhibitor 1A	CDKN1A	18 kDa	488
Q13363	C-Terminal Binding Protein 1	CtBP1	39 kDa	369
P62877	RING-Box 1	RBX1	12 kDa	253
Q99460	Proteasome 26S subunit, non-ATPase 1	PSMD1	106 kDa	181
P24941	Cyclin-Dependent Kinase 2	CDK2	34 kDa	106

Supplementary Table-2. Primers used for qRT-PCR

Gene	Forward	Reverse
CUL1	5’-CAGCATTCAAGTGCTGAGCT-3’	5’-GCTGGCGTAGAAAGCTGTGAAT-3’
CUL2	5’-TGTGCTACTCCACAGACATC-3’	5’-GGTTGTAAGGGTAACAGGTA-3’
CUL3	5’-AGGTGATTGCTGCTATTCTG-3’	5’-ACGTCTCTTCTTCCTTCTGGC-3’
CUL4A	5’-TGTGGCAAAGCACGTGTGCT-3’	5’-TGACGATAGCAGCATCAATCTG-3’
CUL4B	5’-GGAGAACACTGCAGTCATTAG-3’	5’-GCAGCATCAATTTGATACTGTCTG-3’
CUL5	5’-TGCAGTAAGATGTCTCTAGCA-3’	5’-GATGACAGGAAAGCTCTCATG-3’
CUL7	5’-AGACATGCTCAATCAGGCGATTG-3’	5’-CTTGCAGGTACGTCTGAGGT-3’
